# Therapy-Related Acute Promyelocytic Leukemia: A Case Report and a Review of Literature

**DOI:** 10.7759/cureus.42008

**Published:** 2023-07-17

**Authors:** Dawson Foster, Hari K Nair, Katherine Robbins, Nabeel Rajeh

**Affiliations:** 1 Internal Medicine, St. Luke's Hospital, Chesterfield, USA; 2 Hematology Oncology, Saint Louis University School of Medicine, St. Louis, USA; 3 Pathology, Saint Louis University School of Medicine, St. Louis, USA; 4 Internal Medicine-Oncology, Saint Louis University School of Medicine, St. Louis, USA

**Keywords:** acute promyelocytic leukemia (apl), men who have sex with other men (msm), treatment-related, human papillomavirus (hpv), squamous cell, anorectal malignancy

## Abstract

Acute promyelocytic leukemia (APL) is a subgroup of acute myeloid leukemia (AML), and while not a common form of cancer, it does make up a modest portion of acute leukemia. The genetic hallmark of APL is the t(15;17)(q24.1;q21.2) promyelocytic leukemia/retinoic acid receptor alpha (PML/RARA) protein. We present the case of a patient who had undergone prior therapy for stage IIIC squamous cell carcinoma of the anorectal region with 5-fluorouracil, mitomycin C, and radiation and developed therapy-related acute promyelocytic leukemia about 18 months later. We also review the clinical features and management of APL while also highlighting that therapy-related APL, although uncommon, can develop from chemoradiation. The specific diagnosis of therapy-related APL is its own distinct diagnosis, but its treatment remains the same as primary APL.

## Introduction

Acute promyelocytic leukemia (APL) refers to a subgroup of acute myeloid leukemia (AML). Leukemia accounts for a small portion of the overall cancer diagnosis, of which APL does make up a modest portion. APL is a neoplasm of the myeloid lineage, being made up of the promyelocytic cell. Promyelocytes typically contribute up to or greater than 50% of the overall cell population. These promyelocytes have multiple unique features, the most well-known being sticklike aggregates of inclusion bodies known as Auer rods.

If left untreated, APL has a poor median survival, but with quick and appropriate treatment, the threats are significantly limited. The patients have their white blood cell (WBC) count and platelet (PLT) count followed, while their cancer genetics are identified. The genetic hallmark of APL is the t(15;17)(q24.1;q21.2) promyelocytic leukemia/retinoic acid receptor alpha (PML/RARA) fusion protein. Various other genetics have been reported and are important to note; they only make up a small fraction of the reported cases of APL. Multiple risk factors for APL have been identified such as, but not limited to, age, body mass index (BMI), and prior cytotoxic therapies.

Here, we review the topic of APL as its presentation requires quick diagnosis, through monitoring and treatment. APL's presentation, diagnosis, and treatment are outlined in this medical review.

Cases related to prior cytotoxic and radiation therapies are specifically termed therapy-induced acute promyelocytic leukemia, and while the overall number of these cases is low, the incidence has been on the rise as access to cancer treatment has improved while the morbidity and mortality of cancer as a whole have decreased.

In general, therapy-related AML has two common types after alkylating agents. The first type is preceded by myelodysplastic syndrome, which tends to appear 5-10 years post-chemotherapy. This type is commonly associated with monosomy 7 and monosomy 5. The second type occurs usually after two years of topoisomerase inhibitors and is associated with 11q23.

A case of therapy-related APL is also presented as its incidence is likely to increase. The purpose is to highlight key differences and similarities between primary and therapy-related APL and their initiating event. We present the case of a 52-year-old homosexual male who was initially diagnosed and treated for stage IIIC moderately differentiated keratinizing squamous cell carcinoma of the anorectal region with 5-fluorouracil, mitomycin C, and radiation, who was later diagnosed with therapy-related acute promyelocytic leukemia.

## Case presentation

A 52-year-old homosexual male with a history of herpes simplex virus infection, Epstein-Barr virus infection, human papillomavirus infection, and anal condylomata on prophylactic valacyclovir initially presented in December 2018 with six months of intermittent pelvic pain and hematochezia. The patient was approximately 175 cm and weighed 80 kg, resulting in a body mass index of 26.1; of note, the patient was in good physical condition with a great deal of muscle tone. A direct rectal examination demonstrated a mass in the posterior rectum beginning at 3 cm and extending to about 7 cm from the anal verge, with the tumor occupying about one-third of the luminal circumference. A colonoscopy was performed that demonstrated a localized area of moderately thickened mucosal folds at the anus and in the distal rectum. Biopsies were obtained, and pathological examination demonstrated moderately differentiated keratinizing squamous cell carcinoma, with the tumor cells staining positive for p40 and thus confirming the diagnosis.

Staging imaging with computed tomography (CT) of the chest, abdomen, and pelvis and whole-body 18F-fluorodeoxyglucose positron emission tomography (18F-FDG PET) demonstrated the metabolically avid anorectal mass along with perirectal lymph nodes. The patient was staged to have American Joint Committee on Cancer (AJCC) stage IIIC (T3N1M0) squamous cell carcinoma of the anorectal region and was treated with mitomycin C 12 mg/m^2^ intravenously (IV) bolus on day 1 and 5-fluorouracil 750 mg/m^2^ continuous infusion over 120 hours starting on day 1, every 28 days, for two cycles, concurrently with radiation of a total dose of 5400 cGy in 30 fractions to the anorectal region with a boost to the involved lymph nodes, which was completed in March 2019. Posttreatment clinical examination and imaging demonstrated remission.

**Figure 1:**
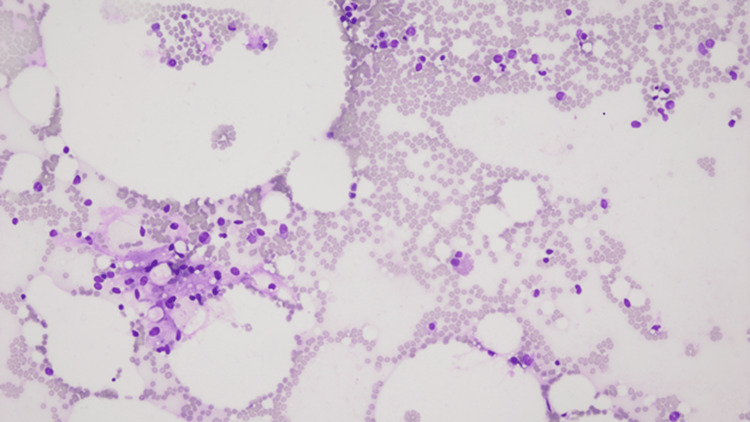
Bone marrow aspirate smear, 200×.

**Figure 2:**
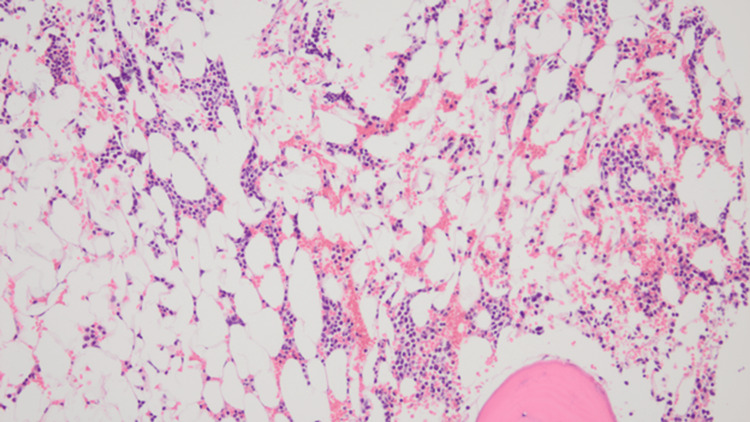
Bone marrow trephine core biopsy, 200×.

**Figure 3:**
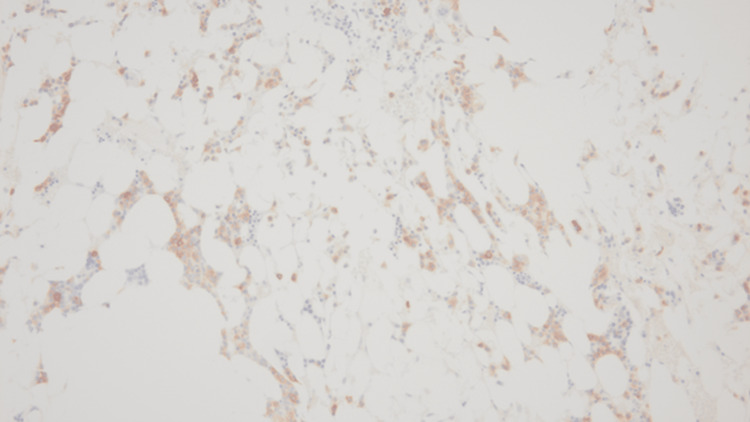
CD117 immunohistochemistry, 200×. CD117: cluster of differentiation 117

**Figure 4:**
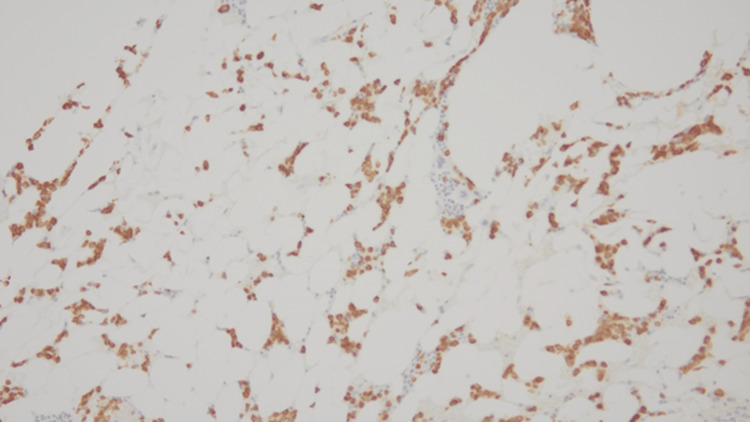
Myeloperoxidase immunohistochemistry, 200×.

In October 2019, during a routine outpatient surveillance evaluation, he was noted to be pancytopenia with mild weakness. His total leukocyte count was 1000 cells/mcL, absolute neutrophil count was 200 cells/mcL, hemoglobin was 11.1 g/dL, and platelet count was 43000 cells/mcL. Nutritional, infectious, and drug-induced etiologies of the pancytopenia were ruled out. A bone marrow examination was performed. The initial diagnostic bone marrow showed adequate spicules that are notably hypocellular. Despite the hypocellularity, 28% of blasts are counted by manual differential count. Blasts are intermediate in size with fine chromatin and relatively scant cytoplasm. No Auer rods are seen. Focal dysplasia is present in the residual hematopoietic elements seen in Figure 1. The bone marrow trephine core biopsy seen in Figure 2 is similarly hypocellular. A few pockets of cells are present that show maturation arrest. In these areas, the predominance of cells shows a cluster of differentiation (CD) 117 (CD117) and myeloperoxidase positivity by immunohistochemistry noted in Figure 3 and Figure 4, respectively. Concurrent fluorescence in situ hybridization (FISH) shows an abnormal result with 30% of cells showing a double fusion of PML/RARA along with one normal signal of each PML and RARA. Karyotype also demonstrated the presence of 46,XY,t(15;17)(q24;q21) in five cells with an additional five cells showing a normal male karyotype (46,XY).

He was diagnosed with treatment-related low-risk acute promyelocytic leukemia and underwent a 28-day induction therapy with all-trans retinoic acid (ATRA) 22.5 mg/m^2^ orally (PO) twice daily (BID) and arsenic trioxide 0.15 mg/kg IV over two hours daily, which was completed in November 2019. He tolerated treatment well with a progressive improvement of his pancytopenia. Bone marrow examination performed after the completion of induction therapy demonstrated negative PML/RARA polymerase chain reaction (PCR).

He then underwent consolidation with all-trans retinoic acid 22.5 mg/m^2^ PO BID for two weeks every four weeks and arsenic trioxide 0.15 mg/kg IV five days a week for four weeks every eight weeks for a total of four cycles, which was completed in July 2020. Bone marrow examinations subsequently demonstrated persistently negative PML/RARA PCR, thus confirming measurable residual disease (MRD)-negative remission. He has since been on clinical and laboratory surveillance every six months and has had normal complete blood counts and negative PML/RARA PCR as of the most recent follow-up in September 2022.

## Discussion

APL refers to a subgroup of AML that was classified formerly as AML-M3 per the French-American-British (FAB) classification system or, currently, as APL with t(15;17)(q24.1;q21.2), which produces the PML-RARA fusion protein [1,2]. While far from the most common type of cancer, APL constitutes 5%-20% of all newly diagnosed cases of acute leukemia in the United States [3-5]. While some data suggests that varying geographic and/or ethnic trends appear higher in the Hispanic population, a cause for this increase in incidence remains undiscovered [6,7]. APL is a disease that appears earlier in adulthood than other acute leukemias, with its incidence increasing in the second decade of life and decreasing and then peaking in the fourth decade of life [8]. The risk factors for APL include elevated body mass index (BMI) and, to a small extent, previous cytotoxic therapies (specifically topoisomerase II inhibitors such as etoposide and doxorubicin) and radiation therapy [9,10].

APL is a neoplasm of the myeloid lineage, specifically the promyelocytic cell. Promyelocytes typically contribute up to or greater than 50% of the overall cell population. Promyelocytes are hypergranular with abundant cytoplasm, circular/oval eccentric nuclei, and occasional indentations. The chromatin is moderately condensed with red/purple cytoplasm and a defining characteristic of Auer rods, which are sticklike aggregates within inclusion bodies [11]. Immunohistochemistry for APL is usually similar to their normal promyelocytic counterparts but can vary based on the APL variant. Markers such as myeloperoxidase, CD13, CD33, and CD79a are commonly positive [11-13].

While the histopathologic and immunohistochemical features are used in the initial diagnosis and helpful in supporting the diagnosis, the genetic features are the hallmark of the APL diagnosis with the t(15;17)(q24.1;q21.2) PML-RARA fusion protein seen in about 90% of the cases [14]. Variants such as, but not limited to, the promyelocytic leukemia zinc finger (PLZF)/RARA t(11;17), nucleophosmin (NPM)/RARA t(5,17), nuclear mitotic apparatus (NuMA)/RARA t(11,17), signal transducer and activator of transcription 5B (STAT5B)/RARA, and interstitial chromosome 17 deletions have been shown to make up the remaining 10% of APL cases. Identifying the chromosomal abnormality is essential as the PLZF/RARA t(11;17) variant is resistant to primary treatment [15-22].

While therapy-related acute promyelocytic leukemia is considered its own distinct category, its treatment remains the same as primary APL. The specific diagnosis of APL requires a history of prior treatment with DNA-damaging agents (cytotoxic chemotherapy and/or radiation therapy), along with the bone marrow involvement of blasts exceeding 20%, the evidence of myeloid origin, and the diagnostic cytogenetics/molecular findings such as t(15;17)(q22;q21.1).

Clinically, the patients with APL present with symptoms similar to that of acute myeloid leukemia. A complete blood count shows pancytopenia leading to weakness, fatigue, shortness of breath, higher risk of infections, and higher bleeding risk. It is important to note that the development of disseminated intravascular coagulation (DIC) and primary hyperfibrinolysis occurs at a higher rate than other AML variants. This is a medical emergency and can cause pulmonary hemorrhage, cerebrovascular hemorrhage, and/or hemorrhagic death in 40% of the patients if left untreated [23-26]. Due to this complication, the close monitoring of laboratory parameters including basic metabolic panel, lactate dehydrogenase (LDH), and uric acid is of crucial importance.

While the cases of therapy-related APL appear low, the incidence appears to be increasing. This is likely due to increasing accessibility to treatment and treatment outcomes [27]. If left untreated, APL has a very short median survival of one month or less. However, with timely diagnosis and treatment, the majority of the patients can be cured. The overall population is not created equal as the patients that are younger than 30 or have a white blood cell count of 10000 or less have a superior event-free survival rate [28]. It is also important to note that elevated age does not predict a poorer outcome [29]. Further delineation into risk stratification was noted by the GIMEMA and PETHEMA trials. These trials categorized risk into low-risk, intermediate-risk, and high-risk groups based on the white blood cell (WBC) count and platelet (PLT) count [30]. See Table 1 for table outline. In summation, the risk of APL is identified from both the cytogenetics and the abovementioned trials.

**Table 1:** Risk stratification of APL delineated by the GIMEMA and PETHEMA trials. APL: acute promyelocytic leukemia

Risk Category	White Blood Count (×10^9^/L)	Platelet Count (×10^9^/L)
Low	≤10	>40
Intermediate	≤10	≤40
High	>10	≤40

In general, therapy-related APL usually presents in two documented ways after an alkylating agent or a topoisomerase inhibitor. The first type is preceded by myelodysplastic syndrome, which tends to appear 5-10 years post-chemotherapy [31-34]. This type is commonly associated with monosomy 5 and monosomy 7. The second type occurs usually after two years of topoisomerase inhibitors and is associated with a 11q23 rearrangement; the addition of radiation therapy has also been noted to increase the therapy-related APL risk [35-39]. Our case highlights a faster time of onset when compared to the usual two-year onset and could indicate the presence of two separate and distinct cancer diagnoses without a direct relation.

The induction treatment aims to reduce the total body leukemia cell population down by multiple factors of 10 by the use of all-trans retinoic acid. All-trans retinoic acid promotes the differentiation of the malignant promyelocytic cell to mature neutrophils. While significant, the treatment of ATRA alone is not substantial enough to induce the desired curative intent, but when used in combination with arsenic trioxide, a complete molecular remission is the norm [40-44]. While the response to the standard treatment is exceptional, there are exceptions. High-risk APL, as discussed above, favors all-trans retinoic acid plus arsenic trioxide and anthracycline or gemtuzumab ozogamicin, while the all-trans retinoic acid plus arsenic trioxide is favored in the low to intermediate risk. It is also important to note that the PLZF/RARA t(11;17) cytogenetics are resistant to all-trans retinoic acid plus arsenic trioxide, requiring the use of chemotherapy as its primary treatment of choice [45-53].

## Conclusions

While therapy-related acute promyelocytic leukemia is considered its own distinct category and is rarer than therapy-related AML, this case and review reiterate that it is important to consider therapy-related acute promyelocytic leukemia in the diagnosis and that treatment remains the same as that of primary APL. Although lower than therapy-related acute promyelocytic leukemia, the incidence of therapy-related acute promyelocytic leukemia appears to be increasing. This is likely due to increasing accessibility to treatment and treatment outcomes. If left untreated, APL has a very short median survival of one month or less. However, with timely diagnosis and treatment, the majority of the patients can be cured.
